# CXCR3/CXCL10 expression in the synovium of children with juvenile idiopathic arthritis

**DOI:** 10.1186/ar1481

**Published:** 2005-01-07

**Authors:** Georgia Martini, Francesco Zulian, Fiorella Calabrese, Marta Bortoli, Monica Facco, Anna Cabrelle, Marialuisa Valente, Franco Zacchello, Carlo Agostini

**Affiliations:** 1 Department of Paediatrics, Padua University School of Medicine, Italy; 2 Pathology Institute, Padua University School of Medicine, Italy; 3 Department of Clinical and Experimental Medicine, Padua University School of Medicine, Italy

**Keywords:** chemokines, CXCL10, juvenile idiopathic arthritis, pathogenesis

## Abstract

The accumulation of T cells in the synovial membrane is the crucial step in the pathophysiology of the inflammatory processes characterizing juvenile idiopathic arthritis (JIA). In this study, we evaluated the expression and the pathogenetic role in oligoarticular JIA of a CXC chemokine involved in the directional migration of activated T cells, i.e. IFNγ-inducible protein 10 (CXCL10) and its receptor, CXCR3. Immunochemistry with an antihuman CXCL10 showed that synovial macrophages, epithelial cells, and endothelial cells bear the chemokine. By flow cytometry and immunochemistry, it has been shown that CXCR3 is expressed at high density by virtually all T lymphocytes isolated from synovial fluid (SF) and infiltrating the synovial membrane. Particularly strongly stained CXCR3^+ ^T cells can be observed close to the luminal space and in the perivascular area. Furthermore, densitometric analysis has revealed that the mRNA levels for CXCR3 are significantly higher in JIA patients than in controls. T cells purified from SF exhibit a definite migratory capability in response to CXCL10. Furthermore, SF exerts significant chemotactic activity on the CXCR3^+ ^T-cell line, and this activity is inhibited by the addition of an anti-CXCL10 neutralizing antibody. Taken together, these data suggest that CXCR3/CXCL10 interactions are involved in the pathophysiology of JIA-associated inflammatory processes, regulating both the activation of T cells and their recruitment into the inflamed synovium.

## Introduction

The trafficking and accumulation of immunocompetent cells are essential components in the pathophysiology of the inflammatory processes. A number of recent data suggest that most of these events are regulated by chemokines, a superfamily of 8–10 kDa molecules that has been divided into four branches (C, CC, CXC, and CXXXC) according to variations in a shared cysteine [1,2]. The current roster approaches more than 50 related proteins. Structural variations of chemokines have been associated with differences in their ability to regulate the trafficking of immune cells during inflammatory disorders. The biological activity of chemokines is mediated by seven-transmembrane-domain, G-protein-coupled receptors classified as C, CC, CXC, or CXXXC chemokine receptors according to the type of chemokine bound. Chemokine receptors are constitutively expressed on some cells, whereas they are inducible on others [3].

Three CXC chemokines (IP-10/CXCL10, Mig/CXCL9, and I-TAC/CXCL11) that are produced in response to IFNγ allow for the accumulation of activated lymphocytes by interacting with a specific receptor (CXCR3) [2]. Although the interactions of chemokine receptors are often characterized by considerable promiscuity, CXCR3 is selective in the recruitment of Th1 cells, B cells, and NK (natural killer) cells but not of nonlymphoid cells. Juvenile idiopathic arthritis (JIA) is characterized by chronic inflammation of the synovium in multiple joints. Early studies of the synovial membrane in JIA have shown the presence of a dense infiltrate of activated T cells clustered around activated dendritic cells, suggesting that lymphocyte recruitment is crucial in the pathogenesis of the disease [4,5]. There is also strong evidence of an up-regulation of IFNγ expression in synovial tissue relative to that in peripheral blood of patients with JIA [6,7], indicating a Th1 type polarization of local inflammatory response. Taken together, these data suggest that lymphocyte-specific CXC chemokines could be involved in the mechanisms promoting the development of inflammatory events in JIA patients.

In this study, using immunohistochemical and molecular studies of tissue sections and flow cytometry evaluation of cells recovered from synovial fluid, we evaluated the role of CXCR3/CXCL10 interactions in the regulation of T-cell migration into the joints of patients with JIA. We have demonstrated the presence of IP-10/CXCL10 in the synovial tissue and its release into the synovial fluid, where it exerts chemotactic activity toward activated CXCR3^+ ^T cells. Taken together, our data suggest that the local production of CXCL10 is involved in the pathophysiology of JIA-associated inflammatory processes.

## Materials and methods

### Study populations

We analyzed synovial tissue from nine patients with oligoarticular JIA who were undergoing arthroscopic synovectomy. All the patients fulfilled the revised criteria for JIA according to the International League of Associations for Rheumatology (ILAR) classification [8] and were managed at the Pediatric Rheumatology Unit of Padua University. The procedure was performed in the case of persistently inflamed joints that did not respond either to systemic anti-inflammatory therapy or to intra-articular steroid injections. In all these patients, gadolinium-enhanced MRI showed marked thickening of the synovial membrane throughout the joint. The patients' mean age at onset of the disease was 70.6 months (range 34–156); the average disease duration at synovectomy was 29.5 months (range 2–60).

As controls, three synovial tissue specimens obtained from children with noninflammatory arthropathy were analyzed by immunochemistry. These subjects had presented with either hexadactylism, bone dysplasia, or bone fracture.

Paired samples of peripheral blood (PB) and synovial fluid (SF) from 20 consecutive patients undergoing intra-articular steroid injection were examined. These patients' mean age at onset of the disease was 77 months (range 13–264) and the mean disease duration was 17 months (range 2–108). Patients who were having systemic anti-inflammatory treatment at the time were excluded from the study.

Since the local ethics committee was not established yet at the beginning of the study, institutional review board approval was not requested, but informed consent was obtained from the parents of all the children included in this study.

### Phenotypic evaluation of lymphocytes from peripheral blood and synovial fluid

The commercially available conjugated or unconjugated monoclonal antibodies used were from the Becton Dickinson (Sunnyvale, CA, USA) and PharMingen (San Diego, CA, USA) series and included CD3, CD4, CD8, CD45R0, CD45RA, and isotype-matched controls. Fluorescein-isothiocyanate-labelled mouse antihuman CXCR3 (R&D Systems Inc, Minneapolis, MN, USA) was also used, and the frequency of PB and SF cells positive for this reagent was determined by flow cytometry as previously reported [9]. Specifically, cells were scored using a FACSCalibur analyzer (Becton Dickinson) and data were processed using the Macintosh CELLQuest software program (Becton Dickinson).

For immunofluorescence analysis, control mouse IgG_1 _and IgG_2a _were obtained from Becton Dickinson.

### Chemotactic activity of synovial fluid

The CXCR3-positive cell line 300-19 (kindly provided by Dr B Moser, Theodor-Kocher Institute, University of Bern, Switzerland) was used to evaluate the chemotactic activity of SF. The cells were grown in RPMI 1640 medium supplemented with 1% glutamine, 5% human serum, 1% kanamycin, and 100 U/ml human recombinant IL-2. Cells were periodically expanded by restimulation with phytohemagglutinin (1 μg/ml) in the presence of irradiated blood mononuclear cells (10:1 ratio of feeder cells : 300-19 cells) and were used for experiments after a culture period of 10 to 14 days.

Cell migration was measured in a 48-well modified Boyden chamber (AC48, Neuro Probe Inc, Gaithersburg, MD, USA). The chamber contains two sections. Chemotactic stimuli were loaded in the bottom section, and cells were put into the top compartment. Polyvinylpyrrolidone-free polycarbonate membranes with 3- to 5-μm pores and coated with fibronectin were placed between the two chamber parts. Only the bottom face of filters was pretreated with fibronectin; this treatment maximizes attachment of migrating cells to filters, increasing their adherence. SF samples or control medium (30 μl) was added to the bottom wells, and 50 μl of 300-19 cells resuspended in RPMI 1640 medium was added to the top wells. The chamber was incubated at 37°C with 5% CO_2 _for 2 hours. The membranes were then removed, washed with PBS on the upper side, fixed, and stained with DiffQuik (Dade AG, Düdingen, Switzerland). Cells were counted in three fields per well at magnification ×800. All assays were performed in triplicate. In blocking experiments, cell suspensions were preincubated before chemotaxis assay for 30 min at 4°C with antihuman IP-10 antibodies at 20 μg/ml. In a few experiments, T cells purified from SF were evaluated for their migratory capability in response to CXCL10 (20 ng/ml and 200 ng/ml, R&D Systems).

Data are expressed as a migration index, which is the ratio between the number of migrating cells in the presence of the stimulus and that in medium alone.

### Immunohistochemical analysis

Expression of CXCR3 and CXCL10 was detected by immunohistochemistry with anti-CXCR3 and anti-IP-10 antibodies, respectively (R&D Systems). Paraffin-embedded sections (4 μm thick) from patients and controls were used for immunostaining with the standard avidin–biotin complex method (Vectastain ABC kit; Vector Laboratories, Burlingame, CA, USA), as previously described [10].

Briefly, for the microwave antigen-retrieval procedure, slides were placed in a 2-L glass beaker containing 0.01 mol/L citrate buffer, pH 5.9, and microwaved at full power (800 W for 5 min, three times) before cooling and equilibration in PBS.

To neutralize endogenous peroxidase activity, we pretreated slides with 3% hydrogen peroxide for 5 min. Primary antibodies were applied at a concentrations of 1:100 for both antibodies (anti-hCXCR3 monoclonal antibody and anti-hIP-10/CXCL10 polyclonal antibody) for 1 hour in a humidified chamber at 37°C. Immunoreactivity was detected using biotinylated secondary antibodies (1:50 rabbit antigoat and 1:1000 goat antirabbit antibodies diluted in PBS–bovine serum albumin buffer) incubated for 45 min, followed by a 30-min incubation with avidin–peroxidase (1:200) and visualized by a 7-min incubation with the use of 0.1% 3,3'-diaminobenzidene tetrahydrochloride as the chromogen. Thereafter the slides were rinsed and washed with PBS for 5 min, and the sections were counterstained with Mayer's hematoxylin. The last steps were performed at room temperature. Control slides were incubated with Tris-buffered saline containing isotype-matched antibodies instead of the primary antibody; they were invariably negative (data not shown). The intensity of antibody staining was classified as strong, moderate, weak, and negative. Parallel control slides were prepared either lacking primary antibody or lacking primary and secondary antibodies, or were stained with normal sera to control for background reactivity.

Immunohistochemistry for the characterization of inflammatory infiltrate, endothelial cells, and synovial cells was carried out using the following monoclonal antibodies CD45 (1:20), CD45RO (1:100), CD20 (1:100), CD68 (1:50), CD4 (1:100), CD8 (1:100), CD31 (1:30) (all from Dako Glostrup, Denmark), and cytokeratin–CAM 5.2 (1:1 Becton Dickinson). The immunoreaction products were developed using the avidin–biotin–peroxidase complex method as described above.

### Confocal microscopy

In order to evaluate the expression of CXCL10 by synovial macrophages, confocal microscopy experiments were performed in three patients with JIA. Paraffined sections were prepared for immunofluorescent labelling. Briefly, primary antibodies against CD68 and IP-10 (diluted 1:50 and 1:1 00, respectively, in PBS with 5 g/L bovine serum albumin and 1 g/L gelatin, respectively) and secondary antibodies (goat antimouse IgG and donkey antigoat IgG) conjugated with Texas red or Alexa 488 (Sigma, Milan, Italy) were used. Double labelling using both antibodies on the same section was performed. Primary antibodies and secondary antibodies were incubated for 1 hour at room temperature. Nuclear staining was carried out with DAPI (4' 6-diamidino-2-phenyindole; Sigma) in PBS. Slides were stored at 4°C and analyzed within 24 hours. As a control, the primary antibody was omitted.

Immunofluorescence was observed with a Leica TCS SL spectral confocal and multiphoton system (Leica, Heidelberg, Germany). We used an argon laser at 488 nm in combination with a helium neon laser at 543 nm to excite the green (CD68) and red (IP-10) fluorochromes simultaneously. Emitted fluorescence was detected with a 505–530-nm band-pass filter for the green signal and a 560-nm long-pass filter for the red signal.

### RT-PCR

RNA was extracted from the tissues using TRIzol reagent (Invitrogen, San Giuliano Milanese, Milan, Italy). The concentration of RNA was estimated by spectrophotometer. The RNA was treated with DNase I (Invitrogen) to remove any genomic DNA that might contaminate the RNA preparations. Complementary DNA (cDNA) was prepared using a synthesis kit (SuperScript II DNA Preamplification System; Invitrogen). A cDNA reaction mixture from 0.1 μg of RNA was used for DNA amplification by PCR. A typical amplification reaction included 2 units of *Taq *polymerase (Takara, Shiga, Japan), 20 pmol of sense and antisense oligonucleotide primers, and 200 μM each of dATP, dCTP, dGTP, and dTTP. Amplification was carried out for 30 cycles: 1 min at 92°C, 1 min at 55°C, and 1 min at 72°C. The amplified DNA was electrophoresed on a 2% agarose gel (Invitrogen), stained with ethidium bromide, visualized under ultraviolet light, and photographed.

The primer sequences used were as follows: for glyceraldehyde-3-phosphate dehydrogenase (GAPDH), 5'-TCC-ATG-ACA-ACT-TTG-GTA-TCG-3' (sense) and 5'-GTC-GCT-GTT-GAA-GTC-AGA-GGA-3' (antisense); for CXCR3, 5'-TTG-ACC-GCT-ACC-TGA-ACA-TA-3' (sense) and 5'-ACG-TCT-ACC-CTG-CTT-TCT-CG-3'. The expected sizes for the cDNA amplicons were as follows: 376 bp for GAPDH, 377 bp for IP-10, and 456 bp for CXCR3. All assays were performed in triplicate.

The number of cycles (30) was chosen to ensure that the amount of products synthesized was proportional to the amount of specific mRNA in the original preparation.

After PCR amplification, PCR products (15 μl) were subjected to electrophoresis on 2% agarose gels containing 0.03 μg/ml ethidium bromide. The quantification of transcript level was carried out by scanning photographs of gels and analyzing the area under peaks, using Quantity one Biorad software. Levels of mRNA expression were normalized by calculating them as a percentage of 3GAPDH mRNA expression levels [11]. The band intensity for 3GAPDH did not differ significantly between experiments.

### Statistical analysis

Data were analyzed with the assistance of the Statistical Analysis System. Data are expressed as means ± standard deviation. Mean values were compared using the ANOVA test.

## Results

### Immunohistochemical analysis of the expression and cellular distribution of CXCL10 in the synovial membrane during JIA

**Figure 1:**
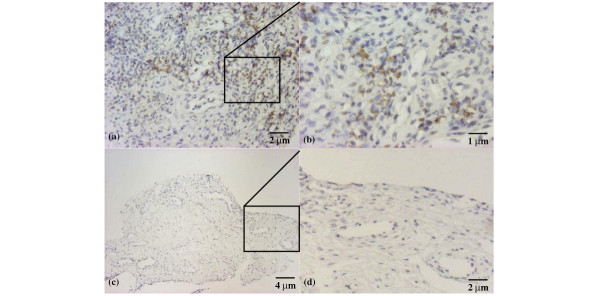
IP-10/CXCL10 expression in the synovium of a patient with juvenile idiopathic arthritis. Few inflammatory cells showing moderate staining; original magnification ×50 **(a)**, ×100 **(b)**. Negative staining in control patient: panoramic view **(c) **(original magnification ×25) and particular view **(d) **(original magnification ×50).

**Figure 2:**
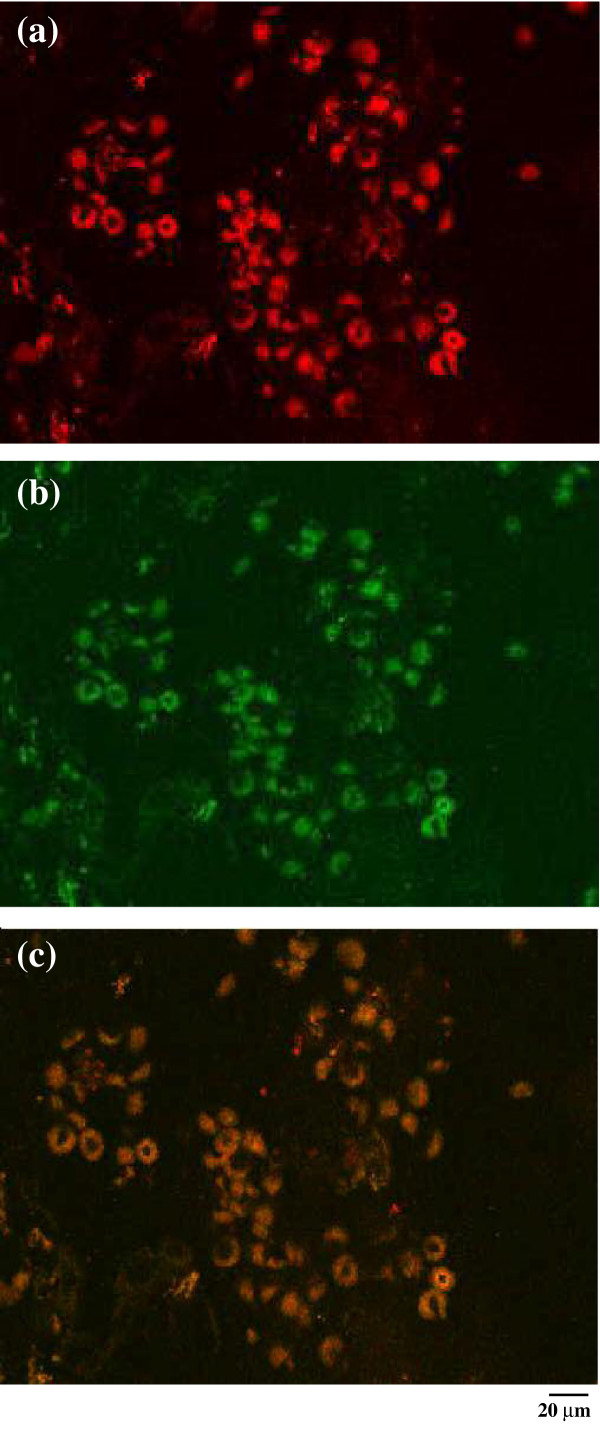
Expression of IP-10/CXCL10 in the synovium of a representative patient with JIA. Immunofluorencence confocal laser scanning microscopy indicates the presence of chemokine IP-10 (red) **(a)**; **(b) **the same cells are shown to be synovial macrophages, as they are marked with CD68 (green). **(c) **The co-localization of IP10 and CD68 by macrophages (brown) is clearly visible. Original magnification ×1000.

Immunohistochemical analysis was used to investigate the pattern of expression of this chemokine in synovial membranes from nine children with JIA and three age-matched controls. All the JIA synovial tissues showed moderate or strong staining for CXCL10 (Table 1). As shown in Fig. 1a and, at higher magnification, in Fig. 1b, CXCL10 was demonstrated on the surface of three types of cells, specifically macrophages, epithelial cells, and endothelial cells, as determined by cell morphology. Most of the IP-10-expressing cells were macrophages. Matched controls revealed no CXCL10 staining (Fig. 1c,d). In order to verify whether macrophages express CXCL10 morphology, data were confirmed by the use of confocal microscopy. As shown in Fig. 2, double staining with CD68 and CXCL10 clearly demonstrated that CD68^+ ^macrophages showed an intense coexpression of the chemokine.

### CXCL10 is present in synovial fluid from patients with JIA and mediates chemotactic activity

**Figure 3:**
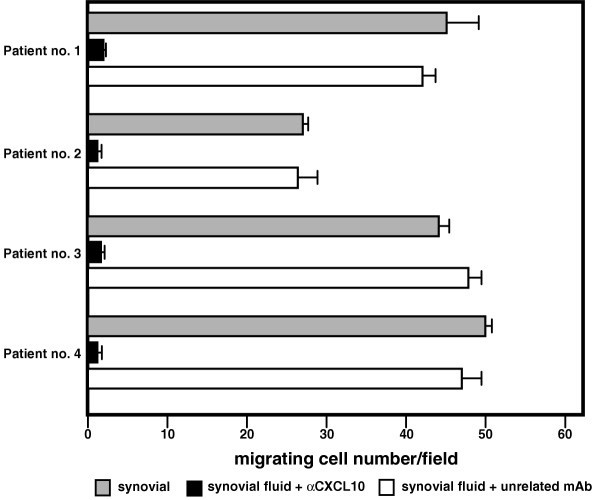
Chemotactic activity of 300-19 cells in the presence of synovial fluid alone (grey bar), synovial fluid with an anti-CXCL10 neutralizing antibody (αCXCL10) (black bar), and synovial fluid with a control antibody (white bar) from four representative patients with juvenile idiopathic arthritis.

**Figure 4:**
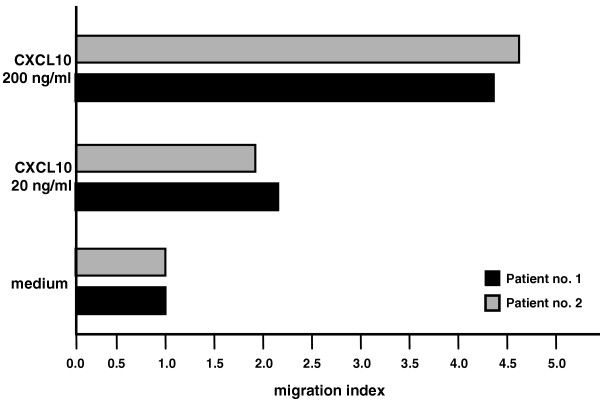
Chemotactic activity migration indices of T cells from synovial fluids of two representative patients with juvenile idiopathic arthritis in the presence of RPMI 1640 medium alone or medium containing CXCL10 at 20 ng/ml or at 200 ng/ml.

To evaluate if CXCL10 is released into the SF and is capable of inducing T-cell migration, the chemotactic activity of supernatants from the SF of four patients with JIA was tested on a T-cell clone expressing high levels of CXCR3 (300-19). As shown in Fig. 3, SF of all the patients we studied exerted significant chemotactic activity on the CXCR3^+ ^T-cell line. The addition of an anti-CXCL10 neutralizing antibody (α CXCL10) but not of a control antibody inhibited chemotactic activities, suggesting the presence of IP-10/CXCL10 in SF and its responsibility in the chemotaxis of CXCR3^+ ^cells. In a second set of experiments, T cells purified from SF exhibited a definite migratory capability per se, which was significantly enhanced in response to CXCL10. Two representative experiments are represented in Fig. 4.

### Immunohistochemical and flow cytometry analysis of the expression of CXCR3 by PB, SF, and synovial-tissue T lymphocytes in JIA

**Figure 5:**
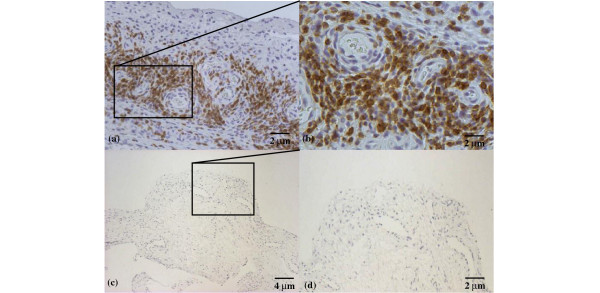
CXCR3 expression in the synovium of a patient with juvenile idiopathic arthritis. Note the marked staining of inflammatory cell infiltrate in the perivascular area [original magnification ×50 **(a)**, ×100 **(b)**]. Negative staining in control patient: panoramic view **(c) **(original magnification ×25) and particular view **(d) **(original magnification ×50).

**Figure 6:**
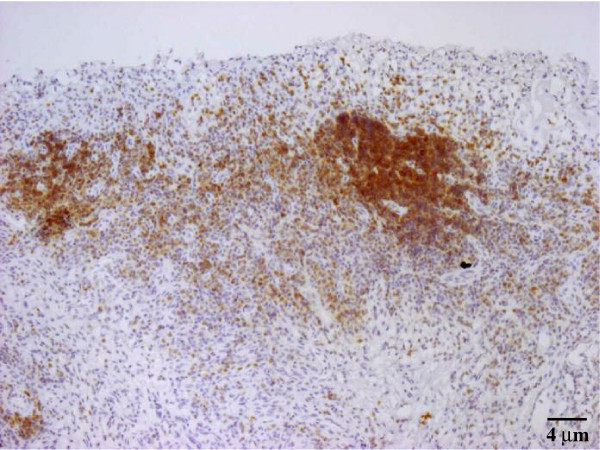
CXCR3 expression in juvenile idiopathic arthritis synovium. A follicular pattern of strongly marked lymphocytes is visible close to the lumen surface. Original magnification ×25.

The possibility that CXCL10 in synovial fluid and membrane might account for the recruitment of CXCR3^+ ^T lymphocytes from the bloodstream to the synovium was investigated by immunohistochemical analysis of the expression of this chemokine receptor. All the JIA patients showed CXCR3-expressing lymphocytes infiltrating the synovium, with strong or moderate staining intensities (see Table 1). Particularly strongly stained cells were observed close to the perivascular area (as in Fig. 5a,b, showing two different magnifications of the same slide). In a few cases, a follicular pattern of strongly marked lymphocytes was visible close to the luminal space (Fig. 6). The control synovial tissues revealed no CXCR3 staining (Fig. 5c,d).

**Figure 7:**
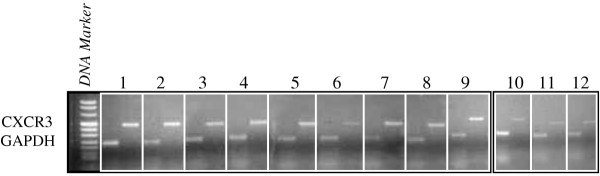
Semiquantitative RT-PCR determination of CXCR3 expression in patients and controls. Unnumbered frame: DNA marker. Representative results of agarose-gel electrophoresis of RT-PCR products of CXCR3 mRNA (456 bp) and glyceraldehyde-3-phosphate dehydrogenase (234 bp) for nine patients (frames 1–9) and three controls (frames 10–12).

Densitometric analysis showed that CXCR3 mRNA levels were significantly higher in patients with JIA than in controls (CXCR3:GADPH ratio 2.25 ± 1.8 vs 0.6 ± 0.49, *P *< 0.05) (Fig. 7).

**Figure 8:**
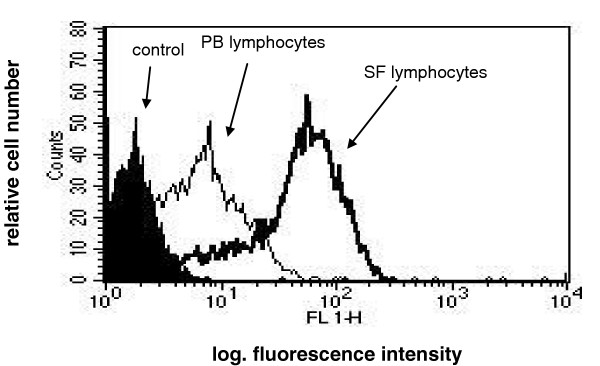
Flow cytometry profile of CXCR3 expression in peripheral blood (PB) and synovial fluid (SF) lymphocytes from patient 3 and a control subject.

Flow cytometry analysis confirmed the selective recruitment of CXCR3^+ ^lymphocytes into the synovium. We analyzed paired samples of PB and SF from 20 children with JIA, and in 18 of these patients, T lymphocytes isolated from the SF showed greater expression of CXCR3 with than did those from PB, both in terms of percentage of positive cells and of the MFI (*P *= 0.01) (Table 2). Flow cytometry profiles for one representative patient are shown in Fig. 8. Taken together, these results strongly suggest a role for the CXCL10 released into the synovial compartment in the accumulation of its selective CXCR3-receptor expressing T cells.

## Discussion

JIA is characterized by a persistent accumulation in the synovial membrane of T lymphocytes most of which express surface markers indicative of activation, such as CD45RO, and a type-1 cytokine profile [4,5]. The cellular infiltrate is defined largely by the composition of locally produced chemokines as well as by the diversity of circulating leukocytes expressing the relevant receptors. Our principal findings are that in JIA, CXCL10/IP-10 is strongly expressed in synovial membranes and is released into synovial fluid (SF), where it exerts a definite chemotactic activity on CXCR3^+ ^T-cell clones and on T cells purified from SF; and that there is an accumulation of CXCR3 expressing T lymphocytes from the bloodstream to the synovial fluid and membrane. These findings suggest a role for CXCL10 in the mechanism of T-cell activation and recruitment into the inflamed synovium.

The high expression of CXCR3 by T cells retrieved from the synovia of patients with JIA might be considered a by-product of the *in vivo *cell hyperactivity of the tissue T-cell compartment in this disease. In fact, recent data clearly indicate that CXCR3 and its ligands become functional on recently activated T cells [12]. After antigenic challenge or in response to stimulation through the T-cell receptor (TCR), T cells express CXCR3, respond with chemotaxis to CXCR3 ligands, and produce IFNγ. Furthermore, in the presence of persistent antigenic stimulations, CXCR3 expression is maintained and poised for rapid up-regulation with reactivation. We and other authors have previously shown that CXCR3/CXCL10 interaction is involved in the pathogenesis of other Th1-mediated processes, such as Crohn's disease and sarcoidosis [13,14]. A similar sequence of events could take place in the synovia of children with JIA. In fact, as previously reported [15], the evaluation of the molecular organization of the TCR revealed that T cells proliferating in children with JIA show a preferential usage of definite TCR gene regions, indicating an ordered immune response in which a specific TCR has been triggered and CXCR3 expression is induced [16].

CXCL10 was expressed by macrophages in synovial membrane of patients with JIA but not of controls. This finding suggests that CXCL10 is part of the matrix of cytokines that regulates the accessory activity of macrophages at sites of inflammatory lesions in the synovial microenvironment. Since large amounts of type 1 inflammatory cytokines, such as IFNγ, tumor necrosis factor α, IL-15, and IL-18, have been detected in JIA synovium [7], it is likely that these cytokines act in concert, sustaining the local proinflammatory responses and up-regulating CXCL10 expression. In turn, since CXCL10 is known to be capable of up-regulating cytokine synthesis in human Th1 cells, it is likely that macrophage-derived chemokines as IL-18 and IL-15 could participate in the maintenance of the default Th1/Tc1 polarization seen during JIA inflammation. It should be noted that, as shown in Fig. 2, anti-IP-10 almost completely inhibited the migration of the CXCR3^+ ^300-19 T cells in response to synovial fluid. Given the ability of I-ITAC/CXCL11 and Mig/CXCL10 to favor T-cell recruitment [17], we are currently investigating whether this non-ERL chemokine may influence entry of T cells into the JIA synovia.

It remains to be established whether synovial endothelial cells express CXCL10 (Fig. 1a). In a previous report it has been shown that human umbilical-vein-derived endothelial cell monolayers stimulated with IFNγ and tumor necrosis factor α produce IP-10/CXCL10, retaining it on their surface, and that this leads to a rapid adhesion of T lymphocytes. This effect was drastically reduced by anti-CXCR3 monoclonal antibody [18]. Furthermore, it is known that unstimulated human umbilical-vein-derived endothelial cells are able to retain IP-10 added exogenously, through binding to cell-surface proteoglycans [19]. Finally, recent data have definitively demonstrated that human endothelial cells may express a previously unrecognized receptor for CXC chemokines named CXCR3B and derived from an alternative splicing of the CXCR3 gene [20]. This receptor shows higher affinity for CXCL10 than classic CXCR3, mediates the inhibition of endothelial-cell growth, and accounts for the known angiostatic capability of CXCL10. Thus, it is possible that nonspecific binding of IP-10 may be responsible for the CXCL10 positivity we observed on endothelial cells. Further studies are in progress to determine whether synovial endothelial cells express CXCR3B *in vivo *and, if this be the case, to determine the putative role of CXCR3B/IP-10 interactions on the balance of angiogenic/angiostatic events in the JIA synovia.

Previous studies on chemokines and their receptors in modulating the recruitment of leukocytes at the sites of inflammation suggested that targeting these molecules with engineered agents might have therapeutic utility in down-modulating inflammatory responses. Results of CXCR3 or IP-10/CXCL10 blockade have already been reported in animal models. Recently, some authors have shown a rapid and marked improvement of adjuvant-induced arthritis in rats treated with IP-10 DNA vaccine [21]. Moreover, anti-mCXCR3 neutralizing antibodies were found to inhibit Th1 lymphocyte recruitment to peripheral inflammatory sites in a mouse model [22]. Further studies are needed in animal models to explore the therapeutic potential of CXCR3- or CXCL10-antagonists, with the ultimate goal of offering new clues for immune intervention in Th1-mediated diseases such as JIA and rheumatoid arthritis.

## Conclusion

Our results provide the first evidence of the functional role of CXCR3/CXCL10 interactions in mediating recruitment of T cells at sites of synovial inflammation in JIA. An in-depth molecular study of mechanisms regulating overexpression of CXCR3/CXCL10 might help in defining the role of these molecules in synovial inflammatory responses, offering new insights into elements controlling the immune response within joints.

## Abbreviations

cDNA = complementary DNA; GAPDH = glyceraldehyde-3-phosphate dehydrogenase; IFNγ = interferon γ ; IL = interleukin; JIA = juvenile idiopathic arthritis; PB = peripheral blood; PBS = phosphate-buffered saline; PCR = polymerase chain reaction; RT-PCR = reverse transcriptase PCR; SF = synovial fluid; TCR = T-cell receptor; Th1 = T helper cell type 1.

## Competing interests

The author(s) declare that they have no competing interests.

## Authors' contributions

GM conceived and coordinated the study and drafted the manuscript. FZ participated in the design of the study. FC performed the immunohistochemistry and helped to draft the manuscript. MB and MF carried out the chemotaxis. AC performed the flow cytometry experiments. MV participated in the immunohistochemistry. FZ participated in the design of the study. CA conceived the study and helped in the draft of the manuscript. All authors read and approved the final manuscript

## Figures and Tables

**Table 1:** CXCR3 and CXCL10 expression in patients with juvenile idiopathic arthritis and controls

Subject no.	Sex	Age at onset (months)	CXCR3	CXCL10
Patients				
1	F	54	++	++
2	F	34	+++	+++
3	F	84	+++	++
4	F	70	+++	++
5	F	65	+++	++
6	F	156	+++	++
7	F	141	+++	+++
8	M	70	+++	++
9	F	42	+++	+++
Controls				
1	F	72	+	+
2	F	36	+	+
3	M	24	--	+

+++, strong; ++, moderate; +, weak; --, negative.

**Table 2:** CXCR3 expression in peripheral blood (PB) and synovial fluid (SF) lymphocytes in five representative patients with juvenile idiopathic arthritis

	Mean fluorescence of CXCR3^a^		
		
Patient no.	In PB cells	In SF cells	D/s^b^
1	35.79	55.8	36.5
2	23.02	77.13	36
3	20.27	48.88	22.4
4	15.84	27.75	34
5	16.44	20.59	20.2

^a^*P *≤ 0.001 in every case. ^b^On the Kolmogorov–Smirnov test; D/s values >10, and *P *values <0.05 were considered significant. D/s is calculated as a function of the number of data; it ranged from 0.5 to 100 and is a measure of the significance of the difference between two distributions.
